# Transcranial electrical stimulation of the prefrontal cortex to boost the hypnosis experience: who benefits most?

**DOI:** 10.3389/fpsyg.2023.1217396

**Published:** 2023-07-14

**Authors:** Rinaldo Livio Perri, Gloria Di Filippo

**Affiliations:** ^1^Department of Psychology, University Niccolò Cusano, Rome, Italy; ^2^De Sanctis Clinical Center (CCDS), Rome, Italy

**Keywords:** hypnosis, hypnotizability, tDCS, prefrontal cortex, consciousness

## Abstract

Many attempts have been made to enhance hypnotizability. The most recent studies adopted the non-invasive brain stimulation to deactivate the dorsolateral prefrontal cortex (DLPFC) during hypnosis, indicating this as a promising approach. However, it is still no clear whether individual factors can predict the effects of stimulation on hypnotizability. In the present study we adopted the phenomenological consciousness inventory (PCI) to retrospectively assess the mental processes during hypnosis and to predict hypnotizability, here defined as “hypnoidal state.” The aim was to investigate the possible role of the hypnotic susceptibility on the efficacy of a validated approach of hypnosis enhancement through cathodal transcranial electrical stimulation (tDCS) of the left DLPFC. Results indicated that the lower hypnoidal state at baseline predicted the greater enhancement after the active tDCS. These findings suggest the subjects with lower hypnotic responsiveness as the best candidates for the tDCS interventions of hypnosis enhancement, at least for the montage targeting the left DLPFC. Neurocognitive underpinnings and clinical implications of the results are discussed.

## Introduction

Hypnotizability refers to the individual’s ability to experience hypnosis and hypnotic suggestions. Depending on the methods of assessment, hypnotizability can be measured in terms of behavioural suggestibility or phenomenological experience of hypnosis. Understanding the relation between hypnotizability and hypnosis is relevant to both basic and clinical research. It is for these reasons that attempts have been made to enhance hypnotizability through pharmacological (e.g., Bryant et al., 2012), psychological (e.g., Spanos, 1986) and magnetic stimulation approaches (Dienes and Hutton, 2013; Coltheart et al., 2018). The most recent findings in this field are those of our group documenting relevant increases of hypnotizability following inhibitory transcranial direct current stimulation (tDCS) of the bilateral (Perri et al., 2022) and unilateral (Perri and Di Filippo, 2023) portions of the dorsolateral prefrontal cortex (DLPFC). These studies were conducted on subjects generally falling in the medium range of hypnotizability, still leaving open questions regarding tDCS effects on different categories of hypnotic responsiveness. In fact, as some investigations documented enhancements for the low hypnotizables or “lows” (e.g., Bryant et al., 2012; Kasos et al., 2018), it is reasonable to hypothesize that possible differential effects may emerge for tDCS interventions as well. In order to test this hypothesis, in the present investigation we maintained the same paradigm of a previous study (Perri and Di Filippo, 2023) with the specific aim to consider the role of baseline hypnotizability on the magnitude of the tDCS effects on hypnotizability. Hypnosis was assessed through the phenomenological consciousness inventory (PCI) which adopts a retrospective phenomenological assessment, or noetic analysis, to quantify the mental processes during hypnosis and to predict hypnotizability, henceforth referred to as the “hypnoidal state” as in the PCI conceptualization (Forbes and Pekala, 1993). In fact, the noetic approach aims to quantify the processes and contents of subjective consciousness via the “snapshot” of the mind as provided by the PCI or similar types of quantitative retrospective inventories (Pekala et al., 2017).

## Materials and methods

### Participants

Thirty-six healthy volunteers participated in this study. They were recruited from the student population at the Niccolò Cusano University and randomly assigned to sham (*N* = 18, 6 males, mean age = 23.5 ± 3.5) and cathodal group (*N* = 18, 7 males, mean age = 23 ± 5.8). The study protocol was approved by the ethical committee of the IRCCS Santa Lucia Foundation (Prot. CE/2024_029) and was in accordance with the ethical standards of the 1964 Declaration of Helsinki. All participants gave their written informed consent before participating in the study.

### tDCS and study design

Direct current was transferred by a saline-soaked pair of surface sponge electrodes (25 cm^2^) and delivered by a battery-driven constant current stimulator in a randomized, sham-controlled protocol. A unilateral extracephalic tDCS montage was adopted with the target electrode over the left DLFPC (F3 site of the 10/20 system) and the return electrode over the right deltoid. The target electrode provided a cathodal stimulation (negative current) delivered by the software-based BrainStim stimulator (EMS srl, Bologna, Italy). The current intensity was gradually increased for 10 s at the beginning of the stimulation session (ramp up), delivered at −2.0 mA for 18 min and decreased for 10 s at the end of the session (ramp down) to diminish its perception. In the sham stimulation, the ramp up was delivered for 10 s until reaching −2.0 mA, the current was transferred for 7 s and was followed by a ramp down lasting 10 s. After 18 min of no-stimulation, the ramp up-ramp down cycle was repeated. Participants were asked to guess the stimulation received (active or sham), but identification was at chance level. Also, potential adverse effects of tDCS were assessed by the experimenter at the end of each session, but none of the participants reported any significant adverse effect.

For all participants, the experiment started with the hypnotic induction and the PCI administration (pre-stimulation condition). Afterwards, tDCS stimulation was provided offline and then the hypnosis-PCI procedure was repeated again (post-stimulation condition). The whole experiment lasted about 110 min including instructions and individual rest time.

### Phenomenological hypnotic assessment: the PCI-HAP

The phenomenological consciousness inventory: hypnotic assessment procedure (PCI-HAP) is a phenomenologically based hypnotic assessment instrument. Administration of the PCI-HAP includes different steps: the pre-assessment, a 20 min standard induction procedure including the hypnotic dream of being on vacation, the post-assessment, and completion of the PCI by the client after the session. The PCI is a retrospective 53-item self-report questionnaire assessing the phenomenological experience in reference to a specific stimulus condition during hypnosis (2 min in which participants were told to sit quietly and “just continue to experience the state you are in right now”; Pekala and Kumar, 2007; Pekala et al., 2010). The PCI explores the phenomenological experience through 14 minor and 12 major dimensions of consciousness (e.g., volitional control, self-awareness, internal dialogue). Moreover, the PCI-HAP provides four major domains including the average total expectancy score (ATES) assessing the hypnotic expectancy; the imagoic suggestibility score (ISS), that is the vividness of visual imagery during the hypnotic dream; the self-perceived hypnotic depth score (sr-HDS), and the *hypnoidal state score* (HSS). The HSS is a measure of “hypnotic state” that correlates about 0.60 (Pekala and Kumar, 1984; Forbes and Pekala, 1993) with scores on the Harvard Group Scale of hypnotic susceptibility (Shor and Orne, 1962). The HHS generates an estimate of Weitzenhoffer’s conceptualization of hypnosis and it is based on a regression equation considering 10 of the PCI (sub)dimensions: the HSS may be the only phenomenological, quantifiable measure of hypnosis available to date (Pekala, 2015; Pekala et al., 2017).

### Statistical analysis

Normal distribution of data was verified using the Shapiro–Wilk test. The major domains of the PCI-HAP (ATES, ISS, sr-HDS and HSS scores) and the subdimensions of the PCI were submitted to 2 × 2 RM-ANOVAs with Group (sham, cathodal) and Session (pre-, post-stimulation) as independent and dependent factor, respectively. Results were corrected for multiple comparisons using the Fisher’s Least Significant Difference (LSD) test, and the effect size was calculated as partial eta squared (*η*^2^*p*; ≥0.01, small effect; ≥0.06, moderate effect; ≥0.14, large effect; Cohen, 2013).

To test for the role of hypnoidal state as a predictor of the tDCS effects on the same construct, simple linear regression analysis was performed with Baseline HSS as independent or explanatory X variable, and Differential HSS (measured as post- minus pre-stimulation HSS) as dependent or response Y variable. The overall *α* level was fixed at 0.05.

## Results

**Figure 1:**
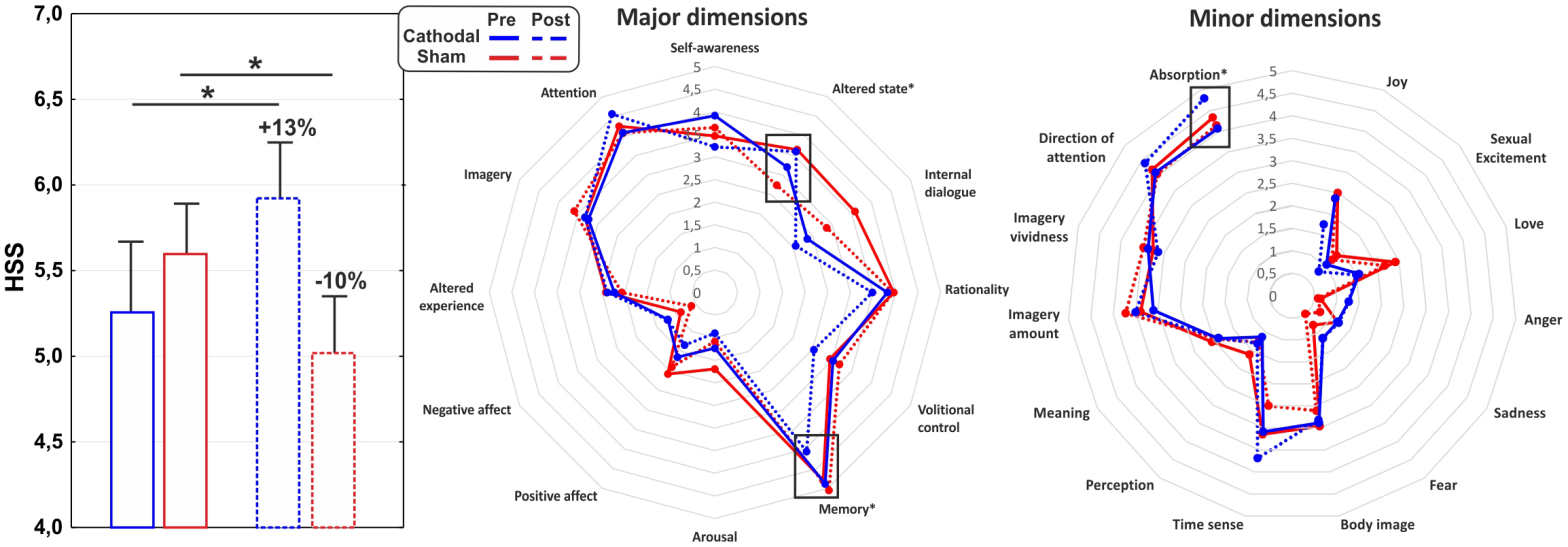
Hypnoidal State Score (HSS) (left), major (middle) and minor dimensions (right) of the PCI for the cathodal and sham group in the pre- and post-tDCS sessions. ^*^p < 0.05.

ANOVAs on the major domains of the PCI-HAP revealed no significant effects for the ISS and sr-HDS scores (all *p*-values > 0.05), while a significant effect of Session emerged for the ATES (*F*_1,34_ = 9.74, *p* < 0.01, *η*^2^*p* = 0.23), indicating greater expectancy for the post-tDCS hypnosis, regardless of the stimulation received. ANOVA on the HSS revealed a significant Group × Session interaction effect (*F*_1,34_ = 9.84, *p* < 0.01, *η*^2^*p* = 0.22): post-hoc analysis documented significant differences between the pre- and the post-stimulation for both the cathodal (HSS increase of about 13%; *p* < 0.05) and the sham group (HSS decrease of about −10%; *p* < 0.05). As for the PCI subdimensions, significant interaction effects emerged for absorption (*F*_1,34_ = 4.15, *p* < 0.05, *η*^2^*p* = 0.12), altered state (*F*_1,34_ = 17.26, *p* < 0.001, *η*^2^*p* = 0.35) and memory (*F*_1,34_ = 7.04, *p* = 0.01, *η*^2^*p* = 0.18). In particular, while Altered State decreased as a repetition effect (i.e., after placebo), absorption and memory were affected by active stimulation in terms of an increase and decrease, respectively. See Figure 1 for a graphical representation of the significant results.

**Figure 2:**
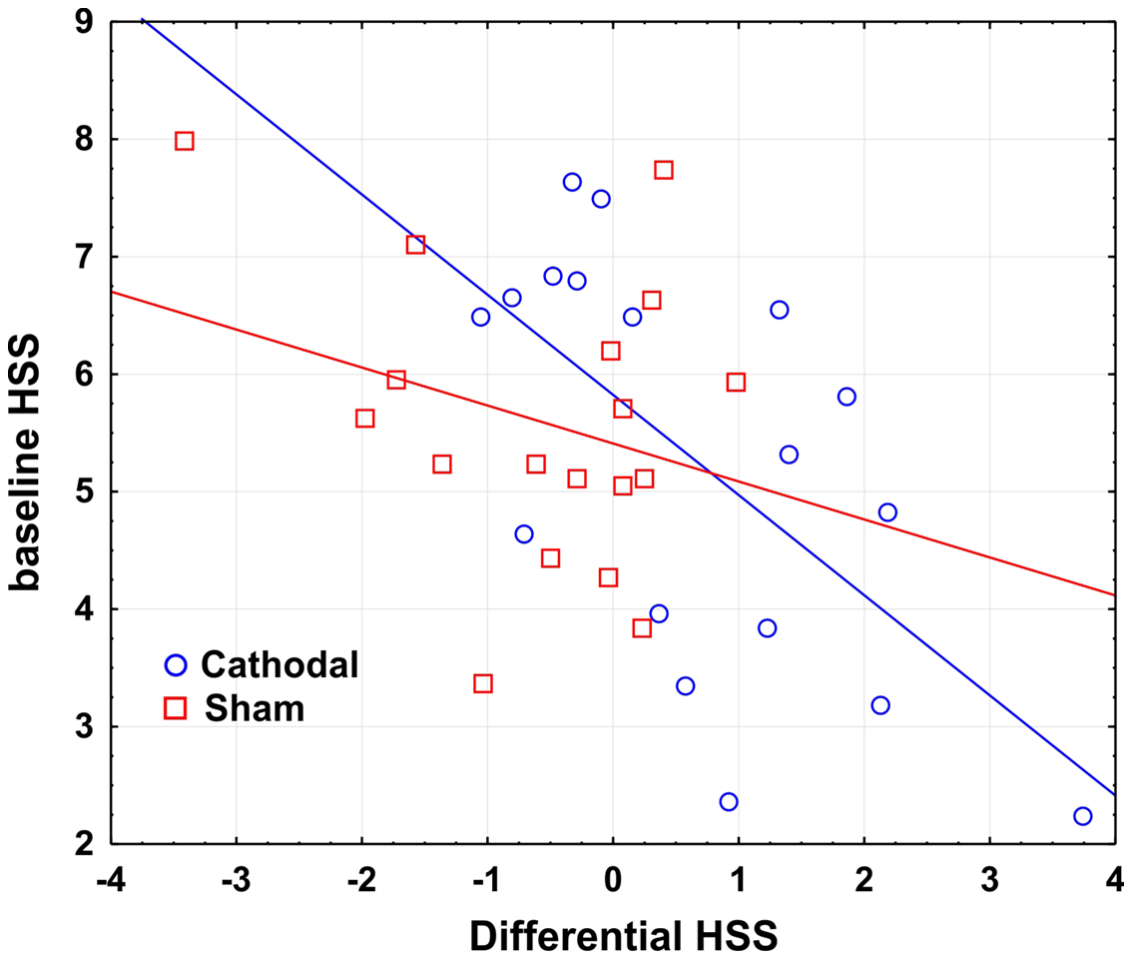
Scatterplot of the regression analysis considering the baseline HSS as a predictor of the tDCS effects on HSS. Differential HSS is calculated as post- minus pre-stimulation HSS score.

Regression analysis indicated the baseline hypnoidal state as a significant predictor of the active tDCS effects on the same construct (*r*^2^ = 0.387, *β* = −0.622, *p* < 0.01), while no significant results emerged for the placebo stimulation (*r*^2^ = 0.8, *β* = −0.28, *p* > 0.05). In other terms, the lower the baseline hypnoidal state the greater the post-cathodal increase of HSS, as depicted in the scatterplot of Figure 2.

In order to deepen the role of baseline hypnoidal state on the tDCS effects, we also provided descriptive statistics on subsamples of subjects selected for their hypnoidal state score. In particular, according to the PCI classification, we adopted the score of 5.00 as a threshold to distinguish subjects with higher (HSS >5.00) and lower (HSS <5.00) responsiveness to hypnosis. This approach yielded the four subsamples described in Table 1: no direct groups comparisons were performed as the small sample size would compromise the statistical reliability.

**Table 1:** Size and scores of the HSS subsamples in the pre- and post-tDCS sessions.

Stimulation	HSS category	n.	pre-tDCS HSS (±SD)	post-tDCS HSS (±SD)
Sham	Medium-lows	5	3.98 (0.63)	3.64 (0.88)
	Medium-highs	13	6.05 (0.97)	5.41 (1.28)
Cathodal	Medium-lows	8	3.55 (0.95)	4.85 (1.22)
	Medium-highs	10	6.61 (0.69)	6.77 (0.78)

**Figure 3:**
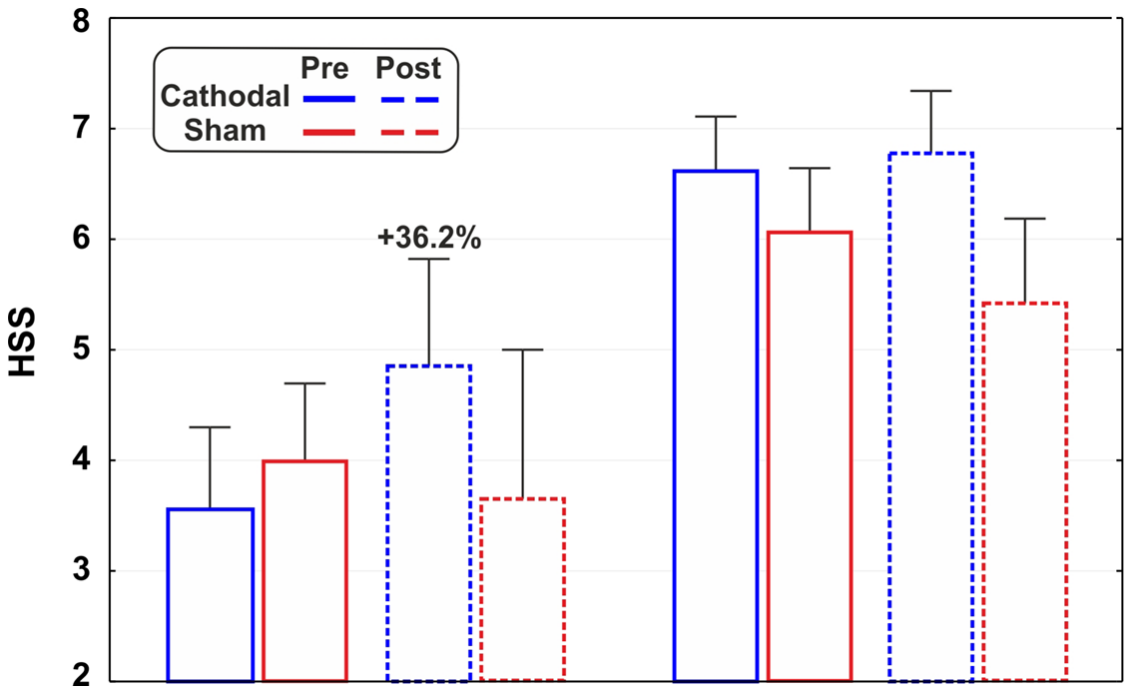
Hypnoidal state scores (HSS) of the subsamples of medium-highs (HSS >5.00) and mediums-lows (HSS <5.00) for active and sham stimulation in the pre- and post-tDCS sessions.

The subsamples were labelled as medium-lows and medium-highs as they fall into the category of mild (HSS score: 3.01–5.00) and moderate (HSS score: 5.01–7.00) hypnoidal state according to the PCI scoring. As reported in Figures 3, a possible variation of the HSS emerged for the cathodal medium-lows as its mean value changed by 36.2% after the active tDCS.

## Discussion

Results of the present research replicated the main findings of previous tDCS studies on hypnosis responsiveness (Perri et al., 2022; Perri and Di Filippo, 2023). Data indicated that cathodal stimulation of the left DLPFC increased the hypnoidal state by 13%, which on the contrary decreased by 10% after the placebo stimulation. The latter data is relevant as well, as it suggests a detrimental learning effect for hypnosis experience which had not yet been detected previously. Obviously, it refers to a very short repetition time as the second hypnosis was administered about 30 min after the end of the previous one: it is still unclear if a similar effect would also occur with hypnosis re-administered over a longer period. It is also noteworthy that the expectancy score (the ATES domain of the PCI-HAP) increased for the post-tDCS hypnosis regardless of the stimulation received. Overall, these data demonstrated that (i) the tDCS apparatus acted *per se* on the individual expectancy (as predictable); (ii) sham was a good placebo as subjects were unaware of the assigned group and its effects were opposite to active stimulation; (iii) hypnosis expectancy did not explain the HSS changes as the hypnoidal score was affected differently by tDCS stimulations.

As for hypnotic susceptibility, the participants reflected the prevailing distribution of the general population, that is mostly a medium level of hypnotizability. In fact, very large recruitments would be needed to select high or low hypnotizables, but assumptions on these “special” categories would not be representative of the general population (Jensen et al., 2017; Perri, 2022; Kekecs et al., 2023). At the opposite, approach of this study allowed us to test the role of baseline hypnoidal state as a predictor of the tDCS effects on the hypnotic experience as assessed by the HSS score of the PCI (Pekala, 2015). In particular, regression analysis demonstrated an inverse relationship between the pre- and the post-tDCS hypnoidal state in the active group. In other terms, the lower hypnoidal state at baseline predicted the greater enhancement after the left DLPFC deactivation. Furthermore, by dividing the whole group into subsamples we directly observed two categories of hypnoidal state, namely the medium-lows and the medium-highs. The descriptive statistics indicated a possible variation of HSS for the subsample of medium-lows (mean HSS = 3.5 on a 0 to 9 scale) as its hypnoidal score changed by 36% after cathodal tDCS. However, due to the small sample size, we cannot support this last observation with statistics and further studies are needed to clarify the effects of tDCS on specific categories of hypnotizability. If confirmed, this finding would indicate individuals with a mild hypnotic susceptibility as the best candidates for the hypnotizability enhancement interventions through tDCS. On the contrary, the reasons for the apparent tDCS ineffectiveness on the medium-highs can be many: for example, (i) it could be possible that a sort of ceiling effect is reached in the hypnotic abilities, making the most skilled individuals insensitive to neurostimulation; (ii) all or at least most individuals have the resources for a good hypnotic experience, but for some reasons the lower hypnotizables still need to uncover them; (iii) tDCS montage of this study is tailored for low hypnotizables as their executive functions are less flexible in achieving the DLPFC deactivation primed by neutral hypnosis (Dienes and Hutton, 2013; Landry et al., 2017; Perri et al., 2020). In order to test these hypotheses and overcome limitations of this study, future investigations with larger samples of highs and lows, and adopting different montages and direct measurements of hypnotizability are needed. In fact, one could test whether stimulating the suggestions-related brain areas would allow greater suggestibility on behavioural measures, and whether the effects are common to all or specific for some hypnotizability categories. Nevertheless, even if efficacy were only confirmed for mild hypnotizables, these findings could have relevant empirical meanings. In fact, the lower hypnotizables would be more in need of boosting interventions when involved in hypnotic procedures: not all the treatments are sensitive to hypnotizability, but a predictor effect of hypnotizability has been documented for clinical outcomes of interventions such as hypnoanalgesia (for a meta-analysis see Thompson et al., 2019).

In conclusion, present study confirms the potential efficacy of the transcranial electrical stimulation as a hypnotizability enhancement procedure, although studies in this field are still in their infancy. Also, the predictor role of HSS prompts future investigations to consider baseline hypnotizability when testing any intervention of hypnosis alteration.

## Data availability statement

The raw data supporting the conclusions of this article will be made available by the authors, without undue reservation.

## Ethics statement

The studies involving human participants were reviewed and approved by Ethical Committee of the IRCCS Santa Lucia Foundation, Rome, Italy. The patients/participants provided their written informed consent to participate in this study.

## Author contributions

RP conceived and designed the study, performed the statistical analysis, and wrote the first draft of the manuscript. GF organized the database and wrote sections of the manuscript. All authors contributed to the article and approved the submitted version.

## Conflict of interest

The authors declare that the research was conducted in the absence of any commercial or financial relationships that could be construed as a potential conflict of interest.

## Publisher’s note

All claims expressed in this article are solely those of the authors and do not necessarily represent those of their affiliated organizations, or those of the publisher, the editors and the reviewers. Any product that may be evaluated in this article, or claim that may be made by its manufacturer, is not guaranteed or endorsed by the publisher.
